# Interrater-Reliabilität der kriteriengeleiteten Beurteilung der Schuldfähigkeit bei paraphilen Störungen

**DOI:** 10.1007/s00115-020-00920-1

**Published:** 2020-05-11

**Authors:** S. Dobbrunz, F. Brunner, J. L. Müller, P. Briken

**Affiliations:** 1 Institut für Sexualforschung, Sexualmedizin und Forensische Psychiatrie, Universitätsklinikum Hamburg-Eppendorf, Martinistraße 52, 20246 Hamburg, Deutschland; 2 Forensische Psychiatrie und Psychotherapie, Georg-August-Universität Göttingen, Göttingen, Deutschland

**Keywords:** Begutachtung, Sexualstraftäter, Steuerungsfähigkeit, Schuldfähigkeit, Sexuelle Devianz, Psychiatric expert opinion, Sexual offender, Accountability, Criminal responsibility, Sexual deviance

## Abstract

**Hintergrund:**

Für die Beurteilung der Schuldfähigkeit bei Sexualdelinquenz ist die Einschätzung des Schweregrades einer paraphilen Störung und der Steuerungsfähigkeit von besonderer Bedeutung. Etablierte Beurteilungskriterien sind unzureichend operationalisiert.

**Ziel und Methoden:**

Vorhandene Kriterien sollen von Experten verschiedener Berufsgruppen hinsichtlich ihrer Reliabilität überprüft werden. Hierzu bewerteten 14 Experten die Kriterien zweier Kriterienkataloge anhand zweier Fallvignetten (siehe Electronic Supplementary Material).

**Ergebnisse und Diskussion:**

Die Interrater-Reliabilität (IRR) bezogen auf die Kriterien von Briken und Müller [3, 4] war höher als die der bisher etablierten Kriterien von Boetticher et al. [2]. Die Auswertung der subjektiven Wichtigkeit der Kriterien beider Beurteilungsskalen zeigte, dass alle Kriterien als mindestens durchschnittlich ausschlaggebend bewertet wurden. Es kann daher zunächst sinnvoll sein, beide Kriterienkataloge für eine höhere Transparenz in den Beurteilungsprozess miteinzubeziehen und dieses Vorgehen in weiteren Studien zu untersuchen.

**Zusatzmaterial online:**

Die Onlineversion dieses Beitrags (10.1007/s00115-020-00920-1) enthält weitere Infomaterialien. Beitrag und Zusatzmaterial stehen Ihnen auf www.springermedizin.de zur Verfügung. Bitte geben Sie dort den Beitragstitel in die Suche ein, das Zusatzmaterial finden Sie beim Beitrag unter „Ergänzende Inhalte“.
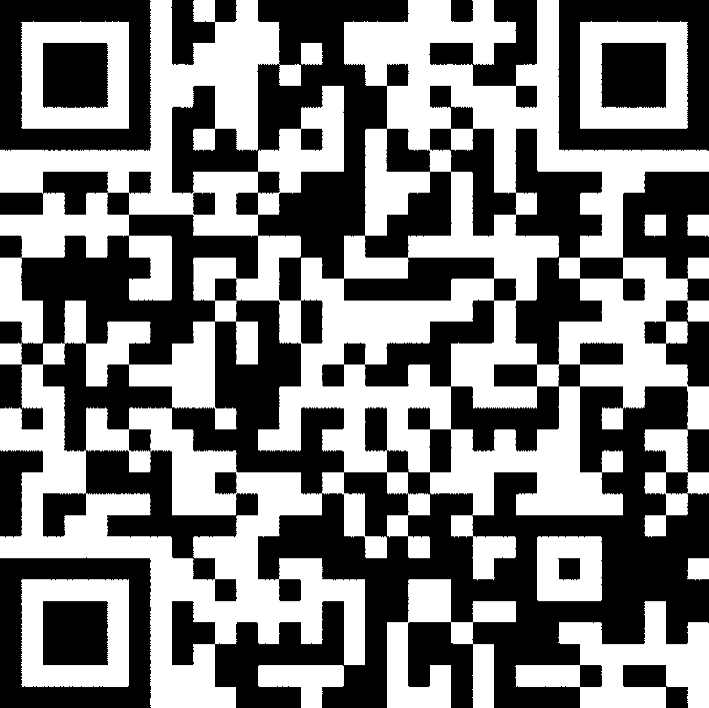

## Abstract (en)

**Background:**

For the assessment of criminal responsibility in the context of sexual delinquency, the evaluation of the severity of a paraphilic disorder and the ability to be controlled are of particular importance. Established assessment criteria are insufficiently operationalized.

**Aim and methods:**

The existing criteria were tested by experts of various professional groups with respect to the reliability. For this purpose, 14 experts assessed the criteria of 2 different sets of criteria based on 2 case vignettes (see electronic supplementary material).

**Results and conclusion:**

The interrater reliability (IRR) with reference to the criteria of Briken and Müller was higher than the previously established criteria of Boetticher et al. The evaluation of the subjective importance of the criteria of both assessment scales showed that all criteria were rated as at least of average importance. It could thus be useful to initially include both sets of criteria for a greater transparency in the assessment process and to investigate this procedure in further studies.

## Hintergrund und Fragestellung

Laut § 20 des Strafgesetzbuches (StGB) „*handelt ohne Schuld, wer bei Begehung der Tat wegen einer krankhaften seelischen Störung, wegen einer tiefgreifenden Bewusstseinsstörung, wegen Schwachsinns oder einer schweren anderen seelischen Abartigkeit unfähig ist, das Unrecht der Tat einzusehen oder nach dieser Einsicht zu handeln“*. „*Ist die Fähigkeit des Täters, das Unrecht der Tat einzusehen oder nach dieser Einsicht zu handeln, aus einem der in § 20 bezeichneten Gründe bei Begehung der Tat erheblich vermindert, so kann die Strafe gemildert werden“ *(§ 21 StGB). Der Schuldbegriff ist im Gesetzestext nicht positiv formuliert. Der Gesetzgeber gibt vielmehr psychische Zustände oder Situationen vor, denen es an Schuld ermangeln kann. Dazu zählen die Unfähigkeit, bei Begehung der Tat das Unrecht der Tat einzusehen oder nach dieser Einsicht zu handeln, aufgrund:

- einer krankhaften seelischen Störung,
- einer tiefgreifenden Bewusstseinsstörung,
- Schwachsinns oder
- einer* schweren anderen seelischen Abartigkeit („SASA“)*.

Die Einschätzung der Schuldfähigkeit folgt dabei einer 2‑stufigen sog. „psychisch-normativen“ Methode [7]. Dabei erfolgt im ersten Schritt eine mögliche Zuordnung zu einem der vier o. g. Eingangskriterien, wobei auch ein Zusammenwirken mehrerer psychischer Störungen in Betracht gezogen und ein Zusammenwirken selbiger somit die Schwelle der ersten Stufe nehmen kann. Im zweiten Schritt werden Auswirkungen der genannten Zustände auf die Einsichts- und Steuerungsfähigkeit betrachtet. Die Frage der Schuldfähigkeit bzw. strafrechtlichen Verantwortlichkeit der einer Straftat beschuldigten Person beantwortet allerdings allein das Gericht. Dabei kann es sich sachverständig beraten lassen. Zu diesem Zwecke können durch das Gericht externe Schuldfähigkeitsgutachten eingeholt werden.

Paraphile Störungen können dabei unter das vierte Eingangsmerkmal der sog. „SASA“ fallen und stellen die Frage einer erheblichen Verminderung der Steuerungsfähigkeit zur Diskussion, was rechtlich eine Dekulpation im Sinne des § 21 StGB zur Folge haben kann [16]. Für den Begutachteten kann das Ergebnis der Schuldfähigkeitsbegutachtung mit erheblichen Folgen verbunden sein, denn eine mögliche Unterbringung in einem psychiatrischen Krankenhaus nach § 63 StGB kann unter bestimmten Umständen lang oder sogar zeitlich unbegrenzt andauern.

Die Prävalenzraten paraphiler Störungen weisen je nach Stichprobe deutliche Unterschiede auf. Besonders aussagekräftig ist die Untersuchung einer österreichischen Population männlicher inhaftierter Sexualstraftäter (*N* = 1346) [5]. Dabei zeigten irgendeine paraphile Störung 43,3 %, eine sexuell sadistische Störung 4,4 %, eine pädophile Störung 34,5 % und eine exhibitionische Störung 2,8 %. Für die Allgemeinbevölkerung hingegen finden sich folgende Lebenszeitprävalenzen für mindestens einmalige paraphile Verhaltensweisen (nicht zu verwechseln mit Störungen): irgendeine paraphile Verhaltensweise 25 %, sexuell sadistische 2,7 %; pädophile 0,9 % und Exhibitionismus 4,3 % [1]. Im Jahre 2009 waren 12 % der Insassen psychiatrischer Krankenhäuser nach § 63 StGB wegen eines Sexualdeliktes untergebracht. Im Jahr 1995 lag dieser Anteil noch bei 21 % [17]. Neben paraphilen Störungen können selbstverständlich auch andere psychiatrische Störungsbilder u. a. aus dem Spektrum der Intelligenzminderungen, Schizophrenien oder auch hirnorganischen Schädigungen mit Sexualdelinquenz im Zusammenhang stehen.

Vorhandene Beurteilungskriterien in Bezug auf das Eingangskriterium der „SASA“ beziehen sich auf heterogene psychische Störungsbilder, vor allem auf Persönlichkeitsstörungen und sind nicht ausschließlich fokussiert auf paraphile Störungen [13–15]. Die so genannten Mindestanforderungen für die Schuldfähigkeitsbegutachtung [2], die von einer interdisziplinären Expertengruppe ausgearbeitet wurden, orientieren sich an aus heterogenen Quellen differierender theoretischer Modelle stammenden Kriterien (z. B. [14]) und den Auswirkungen von Persönlichkeitspathologien auf die Steuerungsfähigkeit. Diese Kriterien sind zusätzlich zu ihrer Nennung nicht weiter operationalisiert, sondern illustrieren psychische Zustände und deren Auswirkungen. Briken und Müller [3] schlugen vor einiger Zeit in dieser Zeitschrift zunächst Kriterien vor, die inhaltlich Schweregrad und Steuerungsfähigkeit abbilden könnten, durch ihre Verwendung in Prognoseinstrumenten bereits standardisiert waren und so möglicherweise die Beurteilerübereinstimmung bei der Einschätzung einer „SASA“ und der hierdurch beeinträchtigten Steuerungsfähigkeit verbessern könnten. Die Autoren wählten dazu 8 Items aus zwei etablierten standardisierten Prognoseverfahren für Sexualstraftäter aus: dem STABLE-2007 [10] und dem ACUTE-2007 [11]. Diese wurden in einem zweiten Schritt von Brunner et al. [4] hinsichtlich ihrer Anwendbarkeit und Interrater-Reliabilität (IRR) untersucht und weiter ausgearbeitet. Diese Pilotstudie zeigte, dass die Kriterien gut anwendbar sind. Neben einer geringen Stichprobengröße macht jedoch die fehlende Einbeziehung der Expertise von juristischer Seite weitere Forschung notwendig. Zudem fehlen weitere Untersuchungen zu Reliabilität und Validität.

Ziel der vorliegenden Arbeit war es erstens, den bereits vorhandenen Kriterienkatalog aus den Mindestanforderungen zur Schuldfähigkeitsbegutachtung [2] und den neu vorgeschlagenen Katalog [3, 4] zur Beurteilung der Schuldfähigkeit bei paraphilen Störungen von verschiedenen Berufsgruppen (PsychiaterInnen, PsychologInnen, RichterInnen) hinsichtlich ihrer Reliabilität zu überprüfen. Zweitens sollte geprüft werden, wie die Übereinstimmung hinsichtlich der abschließend zu beurteilenden SASA und der Steuerungsfähigkeit in Bezug auf die beiden Vignetten war (dies nun nicht mehr getrennt für die beiden Kriterienkataloge). Die eine Vignette stellte einen Fall mit SASA und verminderter Steuerungsfähigkeit dar, die andere einen ohne SASA mit erhaltener Steuerungsfähigkeit. Die Vignetten sollen für weitere Studien verwendet werden und die Beurteilung der hier untersuchten Experten dafür als korrektes Ergebnis gelten, wenn die Beurteilerübereinstimmung dies rechtfertigt. Mittelfristiges Ziel weiterer geplanter Studien in diesem Projekt ist, die Qualität und Transparenz von Begutachtungen zu erhöhen und somit einen substanziellen Mehrwert für die Praxis der Schuldfähigkeitsbegutachtung zu erzielen.

## Studiendesign und Methode

Es wurden aus der Erfahrung der Autoren (SD, PB) zwei prototypische Fallbeschreibungen zweier mutmaßlicher Sexualstraftäter zur Einschätzung der Schuldfähigkeitskriterien entwickelt. Diese stellen konstruierte Fälle dar, wobei der erste auf „keine erheblich verminderte Steuerungsfähigkeit“ und der zweite auf „erheblich verminderte Steuerungsfähigkeit“ hindeutet (Vignetten im Anhang). Auf zu eindeutige Konstruktionen wurde mit dem Ziel verzichtet, möglichst realitätsnahe Fälle zu entwickeln.

Dreißig ExpertInnen, die aufgrund ihrer praktischen und akademischen Erfahrung mit der Schuldfähigkeitsbegutachtung ausgewählt wurden und sich aus drei Berufsgruppen (MedizinerInner, PsychologInnen, JuristInnen) rekrutieren sollten, wurden zur Studienteilnahme eingeladen. Den Teilnehmenden wurden beide Fallvignetten vorgelegt, welche sie mit den zwei Beurteilungsskalen bewerten sollten: (1) Kriterien nach Boetticher et al. [2], (2) Kriterien nach Briken und Müller ([3, 4]; vgl. Tab. 1). Die Beurteilungen der insgesamt 22 Items erfolgten auf einer 3‑stufigen Rating-Skala (liegt nicht/etwas/vollständig vor) und bezüglich der Einschätzung der paraphilen Störungen, „SASA“ und verminderten Steuerungsfähigkeit in Form einer dichotomen Ausprägung (liegt vor/liegt nicht vor). Zudem wurde die Einschätzung der Wichtigkeit/Relevanz der einzelnen Items auf einer 5‑stufigen Skala („überhaupt nicht“ bis „sehr ausschlaggebend“) erfasst. Zur Stichprobenbeschreibung wurden Alter, Geschlecht, Beruf und die durchschnittliche Anzahl von Schuldfähigkeitsgutachten, bzw. bei JuristInnen Fälle, die mit einer Begutachtung beauftragt wurden, bezogen auf die letzten 5 Jahre, erfasst. In Form eines offenen Antwortformats konnten ergänzende Hinweise zur Bewertung der einzelnen Items angegeben werden.

**Table 1:** 

Kriterienliste	Unterskala	Items	Krippendorff α	Verbale Einschätzung nach Krippendorff	Verbale Einschätzung nach Margraf und Fehm
Boetticher et al. [2]	Einschätzung der „SASA“	Die Paraphilie bestimmt weitgehend die Sexualität des Probanden	0,168	Ungenügend	Ungenügend
		Die Paraphilie wird als ich-fremd („ich-dyston“) wahrgenommen und damit ausgeblendet	0,089	Ungenügend	Ungenügend
		Die Paraphilie ist in ihrer Dynamik progredient	0,067	Ungenügend	Ungenügend
		Dem Probanden stehen keine bzw. kaum andere Möglichkeiten zur Verfügung, sich sexuell zu befriedigen	0,040	Ungenügend	Ungenügend
Briken und Müller [3, 4]	Einschätzung der „SASA“	Paraphile sexuelle Interessen	Eine Berechnung des Koeffizienten Krippendorffs α ist aufgrund fehlender Merkmalsvarianz nicht möglich. Es liegt jedoch eine nahezu perfekte prozentuale Übereinstimmung vor	Nahezu perfekt	Nahezu perfekt
		Sexuelle Dranghaftigkeit/Überbeschäftigung	0,572	Ungenügend	Zufriedenstellend
		Sex als Copingstrategie	0,391	Ungenügend	Ungenügend
		Defizite, stabile Beziehungen aufzubauen	0,793	Moderat	Gut
		Allgemeine soziale Zurückweisung/Einsamkeit	0,811	Zuverlässig	Gut
Boetticher et al. [2]	Einschätzung der Steuerungsfähigkeit	Konflikthafte Zuspitzung und emotionale Labilisierung vor der verfahrensgegenständlichen Tat	0,722	Moderat	Gut
		Tatdurchführung auch in sozial stark kontrollierten Situationen	0,309	Ungenügend	Ungenügend
		Abrupter, impulsiver Tatablauf	0,213	Ungenügend	Ungenügend
		Archaisch, destruktiver Tatablauf, ritualisiert wirkend, Außenreize werden ausgeblendet	0,565	Ungenügend	Zufriedenstellend
		Konstellative Faktoren (Substanzintoxikation, Komorbiditäten)	0,145	Ungenügend	Ungenügend
		Hinweise auf Tatvorbereitungen und ein planmäßiges Vorgehen	0,041	Ungenügend	Ungenügend
		Fähigkeit, zu warten, und/oder ein lang hingezogenes Tatgeschehen	0	Ungenügend	Ungenügend
		Komplexer Tatablauf in Etappen	0,049	Ungenügend	Ungenügend
		Vorsorge gegen Entdeckung	0,237	Ungenügend	Ungenügend
		Möglichkeit, sich in vergleichbaren Situationen anders zu verhalten	0,268	Ungenügend	Ungenügend
Briken und Müller [3, 4]	Einschätzung der Steuerungsfähigkeit	Dranghaftigkeit	0,524	Ungenügend	Zufriedenstellend
		Emotionaler Zusammenbruch	0,596	Ungenügend	Zufriedenstellend
		Zusammenbruch sozialer Unterstützung	0,591	Ungenügend	Zufriedenstellend

Zur Bestimmung der IRR wurde Krippendorffs α [8] und, soweit möglich, Fleiss κ [6] berechnet. Die Berechnungen wurden mit der Statistiksoftware *R* durchgeführt. Die Interpretationsbereiche von Krippendorffs α, die als sehr konservativ einzuschätzen sind, gestalten sich wie folgt: *α* ≥ 0,800: zuverlässige Übereinstimmung, 0,800 > *α* ≥ 0,667: moderat, vorläufige Schlussfolgerungen sind möglich, *α* < 0,667: Daten sind zu verwerfen. Die Interpretationsbereiche von Margraf und Fehm [12] fallen liberaler aus. Die Autoren schlagen für die klinische Diagnostik vor, Werte ab 0,50 als zufriedenstellend und Werte ab 0,70 als gut zu betrachten. Zur Interpretation der Koeffizienten Fleiss κ wird die Einteilung nach Landis und Koch [9] gewählt: Werte <0: keine Übereinstimmung, 0–0,20: geringe Übereinstimmung, 0,21–0,40: befriedigende Übereinstimmung, 0,41–0,60: moderate Übereinstimmung, 0,61–0,80: substanzielle Übereinstimmung und 0,81–1: fast perfekte Übereinstimmung.

## Ergebnisse

Es nahmen 14 (ausschließlich Männer) der insgesamt 30 angeschriebenen ExpertInnen teil: 7 Psychiater, 5 Richter und 2 Psychologen im Alter von durchschnittlich 60,57 Jahren (Range: 43–75 Jahre). In den letzten 5 Jahren haben diese durchschnittlich 30,51 Schuldfähigkeitsgutachten (Range: 0,2–160) angefertigt bzw. bearbeitet.

In der Tab. 1 sind die IRR der Items der beiden Kriterienkataloge und die dazugehörigen Interpretationsbereiche dargestellt.

Die IRR bezogen auf die Kriterien von Briken und Müller [3, 4] fielen höher aus als die IRR der Kriterien von Boetticher et al. [2]. Lediglich bei den beiden Items „konflikthafte Zuspitzung und emotionale Labilisierung vor der verfahrensgegenständlichen Tat“ und „archaisch, destruktiver Tatablauf, ritualisiert wirkend, Außenreize werden ausgeblendet“ ergaben sich ähnlich hohe Reliabilitätswerte wie bei den Items von Briken und Müller [3, 4]. Werte der IRR von mindestens 0,5 konnten dabei bei insgesamt 9 Items gefunden werden, darunter bei 2 der insgesamt 14 Kriterien (14,3 %) von Boetticher et al. [2] und bei 7 der insgesamt 8 Kriterien (87,5 %) von Briken und Müller [3, 4], und zwar bei: „paraphile sexuelle Interessen“, „sexuelle Dranghaftigkeit/Überbeschäftigung“, „defizite, stabile Beziehungen aufzubauen“, „allgemeine soziale Zurückweisung/Einsamkeit“, „Dranghaftigkeit“, „emotionaler Zusammenbruch“ und „Zusammenbruch sozialer Unterstützung“.

Der Tab. 2 sind die IRR und verbalen Interpretationsbereiche der Items „Paraphilie“, „SASA“ und „verminderte Steuerungsfähigkeit“ zu entnehmen. Eine Differenzierung nach beiden Kriterienkataloge fand hier nicht mehr statt, sondern es wurde die Übereinstimmung der abschließenden Einschätzung zwischen den 14 Experten in Bezug auf die beiden Fälle berechnet.

**Table 2:** 

Zu beurteilendes Kriterium	Krippendorffs α	Fleiss κ	Verbale Einschätzung nach Krippendorff	Verbale Einschätzung nach Landis und Koch
Vorliegen einer paraphilen Störung bzw. Störung der Sexualpräferenz	1	Eine Berechnung des Koeffizienten Fleiss κ ist aufgrund fehlender Merkmalsvarianz nicht möglich. Es liegt ein perfekte prozentuale Übereinstimmung vor	Perfekt	Perfekt
Vorliegen einer „SASA“	0,732	0,731	Moderat	Substanziell
Erheblich verminderte Steuerungsfähigkeit	0,592	0,591	Ungenügend	Moderat

Hinsichtlich der Frage nach dem Vorliegen einer paraphilen Störung ist die Beurteilerübereinstimmung perfekt. Bezüglich des Vorliegens einer „SASA“ lag eine moderate bzw. substanzielle Übereinstimmung vor. Bezogen auf das Vorliegen einer „erheblich verminderten Steuerungsfähigkeit“ kann – je nach Beurteilungsgrundlage (Krippendorff bzw. Landis und Koch) – lediglich von einer ungenügenden bzw. moderaten Übereinstimmung ausgegangen werden.

Die Auswertung der subjektiven Wichtigkeit/Relevanz der 22 Items der beiden Beurteilungsskalen hinsichtlich der Zielvariablen „SASA“ ist in Tab. 3﻿ und bezüglich der verminderten Steuerungsfähigkeit in Tab. 4 dargestellt. Von den Items hinsichtlich der Zielvariablen „SASA“ wurden 2 der 9 Items im Mittel als durchschnittlich ausschlaggebend bewertet und 7 als ausschlaggebend. Hinsichtlich der Zielvariablen „verminderte Steuerungsfähigkeit“ wurden 6 der 13 Items im Mittel als durchschnittlich ausschlaggebend (3. Kategorie auf einer 5‑stufigen Skala) und 6 als ausschlaggebend (4. Kategorie auf einer 5‑stufigen Skala) bewertet.

**Table 3:** 

Kriterienliste	Items	*Mittelwert* Vignette 1 Vignette 2 Gesamt	*Streuung (SD)* Vignette 1 Vignette 2 Gesamt	*Minimum* Vignette 1 Vignette 2 Gesamt	*Maximum* Vignette 1 Vignette 2 Gesamt
Boetticher et al. [2]	Die Paraphilie bestimmt weitgehend die Sexualität des Probanden	3,36 4,57 3,96	1,08 0,85 1,14	2 2 2	5 5 5
	Die Paraphilie wird als ich-fremd („ich-dyston“) wahrgenommen und damit ausgeblendet	2,43 3,14 2,79	1,16 1,29 1,26	1 1 1	5 5 5
	Die Paraphilie ist in ihrer Dynamik progredient	3,86 4,50 4,18	1,35 0,76 1,12	1 3 1	5 5 5
	Dem Probanden stehen keine bzw. kaum andere Möglichkeiten zur Verfügung, sich sexuell zu befriedigen	3,36 4,00 3,68	1,08 1,04 1,09	1 2 1	5 5 5
Briken und Müller [3, 4]	Paraphile sexuelle Interessen	3,29 3,93 3,61	0,99 1,00 1,03	2 2 2	5 5 5
	Sexuelle Dranghaftigkeit/Überbeschäftigung	3,86 4,57 4,21	0,86 0,51 0,79	2 4 2	5 5 5
	Sex als Copingstrategie	3,29 3,64 3,46	1,07 1,28 1,17	1 1 1	5 5 5
	Defizite, stabile Beziehungen aufzubauen	3,43 3,86 3,64	1,16 0,86 1,03	2 2 2	5 5 5
	Allgemeine soziale Zurückweisung/Einsamkeit	3,29 3,86 3,57	1,14 0,95 1,07	2 2 2	5 5 5

**Table 4:** 

Kriterienliste	Items	*Mittelwert* Vignette 1 Vignette 2 gesamt	*Streuung (SD)* Vignette 1 Vignette 2 gesamt	*Minimum* Vignette 1 Vignette 2 gesamt	*Maximum* Vignette 1 Vignette 2 gesamt
Boetticher et al. [2]	Konflikthafte Zuspitzung und emotionale Labilisierung vor der verfahrensgegenständlichen Tat	3,50 4,14 3,82	1,56 0,95 1,31	1 3 1	5 5 5
	Tatdurchführung auch in sozial stark kontrollierten Situationen	3,36 3,86 3,61	1,69 1,17 1,45	1 2 1	5 5 5
	Abrupter, impulsiver Tatablauf	2,79 3,50 3,14	1,67 1,45 1,58	1 1 1	5 5 5
	Archaisch, destruktiver Tatablauf, ritualisiert wirkend, Außenreize werden ausgeblendet	3,07 4,07 3,57	1,59 1,14 1,45	1 2 1	5 5 5
	Konstellative Faktoren (Substanzintoxikation, Komorbiditäten)	3,00 3,21 3,11	1,48 1,53 1,47	1 1 1	5 5 5
	Hinweise auf Tatvorbereitungen und ein planmäßiges Vorgehen	3,71 3,71 3,71	1,38 1,14 1,24	1 2 1	5 5 5
	Fähigkeit, zu warten, und/oder ein lang hingezogenes Tatgeschehen	3,79 3,07 3,43	1,53 1,27 1,43	1 1 1	5 5 5
	Komplexer Tatablauf in Etappen	2,93 2,93 2,93	1,44 1,33 1,36	1 1 1	5 5 5
	Vorsorge gegen Entdeckung	3,71 3,64 3,68	1,33 1,47 1,36	1 1 1	5 5 5
	Möglichkeit, sich in vergleichbaren Situationen anders zu verhalten	4,07 2,93 3,50	1,00 1,21 1,23	1 2 1	5 5 5
Briken und Müller [3, 4]	Dranghaftigkeit	3,93 4,57 4,25	1,27 0,65 1,04	2 3 2	5 5 5
	Emotionaler Zusammenbruch	3,36 3,36 3,36	1,55 1,08 1,31	1 1 1	5 5 5
	Zusammenbruch sozialer Unterstützung	3,29 3,64 3,46	1,54 0,93 1,26	1 2 1	5 5 5

Von der Möglichkeit im Rahmen eines offenen Antwortformats ergänzende Hinweise zur Bewertung der einzelnen Items vorzunehmen bzw. anzugeben, welche weiteren Kriterien für Experten in Betracht kommen, machten 3 der 14 Experten Gebrauch. Zwei Experten bemängelten die Fallvignetten (unzureichende Angaben zu möglichen Komorbiditäten; „schablonenhaft“). Der dritte Experte gab an, dass nicht nur das Vorhandensein bestimmter Items, sondern auch das Fehlen selbiger für die Entscheidung relevant sein kann.

## Diskussion

Die Interrater-Reliabilitäten bezogen auf die Kriterien von Briken und Müller [3, 4] fielen in dieser Untersuchung höher aus als die IRR der Kriterien von Boetticher et al. [2]. Mögliche Gründe hierfür könnten sein, dass die Items von Briken und Müller [3, 4], im Gegensatz zu den Kriterien von Boetticher et al. [2], ausführlich operationalisiert sind und somit weniger Inkongruenzen im Verständnis der einzelnen Items bestehen könnten. Je präziser ein Merkmal beschrieben ist, desto höher fällt in der Regel die IRR aus [18]. Dieser Argumentation könnte entgegenstehen, dass die 14 Experten auch ohne ausführliche Operationalisierung ein genaues Verständnis der einzelnen Kriterien von Boetticher et al. [2] haben könnten. Bei immerhin 13 der insgesamt 22 Items, bezogen auf beide Kriterienkataloge, fiel die IRR aber ungenügend aus. Dies traf auf 12 Kriterien von Boetticher et al. [2] und ein Kriterium von Briken und Müller [3, 4] zu. Ursachen hierfür könnten wiederum in möglichen Inkongruenzen im Verständnis der einzelnen Items liegen. Zudem ist es denkbar, dass bei den einzelnen Experten unterschiedliche Schwellen hinsichtlich der Einschätzung der einzelnen Kriterien vorliegen. Außerdem handelt es sich bei den Kriterien um von ihrer Natur her seltene und schwer zu erfassende Ereignisse, die aber, wenn sie auftreten, von besonderem Interesse sind. Folglich wird darauf hingewiesen, dass einzelne Items der Kriterienkataloge nicht überbewertet werden sollten, sondern, dass man möglichst viele Kriterien zur Entscheidungsfindung heranziehen sollte. Als Möglichkeiten zur Verbesserung der IRR kommen neben einer möglichst präzisen Definition der einzelnen Kriterien, die Sicherstellung der fachlichen Expertise – beispielsweise durch Weiterbildungen oder Rater-Trainings – und durch Feedback und Austausch über die Ratings anderer ExpertInnen – z. B. im Rahmen von Intervisionen, in Betracht.

Die IRR hinsichtlich des Vorliegens einer „SASA“ lag in einem moderaten bzw. substanziellen Bereich. Bezogen auf das Vorliegen einer „erheblich verminderten Steuerungsfähigkeit“ – welches das zentrale Kriterium hinsichtlich der Beurteilung der Schuldfähigkeit darstellt – konnte von einer ungenügenden bzw. moderaten Übereinstimmung ausgegangen werden. Dies mag nicht sonderlich vielversprechend klingen, dürfte jedoch durch die Tatsache relativiert werden, dass nur 3 der insgesamt 28 Beurteilungen (14 Beurteiler für 2 Vignetten) abweichend ausfielen. Möglichweise geben die Ergebnisse der Übereinstimmungen hinsichtlich der „SASA“ und der „erheblich verminderten Steuerungsfähigkeit“ auch einen Hinweis darauf, dass es beiden Konzepten einer präzisen psychiatrischen Definition ermangelt.

Die Auswertung der subjektiven Wichtigkeit/Relevanz der 22 Kriterien der beiden Beurteilungsskalen zeigte, dass alle Kriterien im Mittel als mindestens durchschnittlich ausschlaggebend bewertet wurden und somit davon auszugehen ist, dass offensichtlich allen Kriterien ein Beitrag zur finalen Entscheidung (hinsichtlich der Zuordnung zur „SASA“ bzw. „erheblich verminderten Steuerungsfähigkeit“) zuzuschreiben ist.

Limitierend muss darauf hingewiesen werden, dass sich die Bewertungen auf zwei konstruierte Fallvignetten (siehe Electronic Supplementary Material) bezogen, die aus Gründen der Zeitökonomie auch für die Verwendung zukünftiger Studien verhältnismäßig kurz und somit inhaltlich nicht erschöpfend gehalten wurden, in jedem Fall keinen Anspruch auf Vollständigkeit erheben konnten. Des Weiteren fehlte den Experten der persönliche Eindruck des Probanden, wie es in Form einer Exploration im Rahmen einer psychologischen/psychiatrischen Begutachtung oder einer möglichen Hauptverhandlung vor Gericht normalerweise der Fall ist.

Es bedarf weiterer Forschung hinsichtlich der Validität beider Kriterienkataloge, die in bereits laufenden Folgestudien durch die Autoren untersucht wird und dann in einem Expertengremium weiter diskutiert und ausgearbeitet werden soll.

## Fazit für die Praxis

- Es kann derzeit sinnvoll sein, beide Kriterienkataloge für eine höhere Transparenz in den Beurteilungsprozess miteinzubeziehen und dieses Vorgehen in der Forschung weiter zu untersuchen.
- Die vorgeschlagenen Kriterien sind im Begutachtungsprozess jedoch sicher nicht als alleinige Beurteilungsquelle anzusehen.

## Caption Electronic Supplementary Material




