# Supplementary material for: Interrater reliability of the criteria-based assessment of criminal responsibility in paraphilic disorders
Source: Nervenarzt. 2020 May 11;92(1):1–8. [Article in German] doi: 10.1007/s00115-020-00920-1 (PMC7808990; doi:10.1007/s00115-020-00920-1)
Supplement: Supplementary file 1 [file 115_2020_920_MOESM1_ESM.docx]

Fallvignette 1:

Der Angeklagte Herr Z. ist 46 Jahre alt (*12.01.1972). Ihm wird zur Last gelegt, den 4-jährigen Jonas, den Sohn seiner Lebensgefährtin über einen Zeitraum von circa 2 Jahren hinweg wiederholt sexuell missbraucht zu haben. Anhand von Foto- und Videoaufnahmen, die Herr Z. während der sexuellen Handlungen angefertigt habe, seien insgesamt 38 Einzeltaten zuordbar. Herr Z. habe an dem Jungen Oralverkehr durchgeführt und sei auch anal in ihn eingedrungen, dabei habe der Junge teilweise auch geschrien. Herr Z. habe sich während der Tathandlungen jeweils allein mit Jonas in der gemeinsamen Wohnung befunden, hierauf habe er akribisch geachtet. Die Zeugenbefragungen der Lebensgefährtin und Schwester hätten ergeben, dass diese die Anschuldigungen für unvorstellbar halten würden. Die Lebensgefährtin habe zudem angegeben, dass sie zwar schon immer - in der insgesamt 2 ½–jährigen Beziehung zu Herrn Z. - den Eindruck gehabt habe, dass er wenig Interesse an gemeinsamen sexuellen Aktivitäten habe, dieses Verhalten aber mit seiner beruflichen Überlastung erklärt. In den letzten Monaten der Beziehung habe eigentlich gar kein Geschlechtsverkehr mehr stattgefunden. Herr Z. habe vor wenigen Wochen seinen Job als Gebäudereiniger verloren.

In der Beschuldigtenvernehmung der Kriminalpolizei gab Herr Z. u. a. an, dass er zunächst damit begonnen habe den Jungen zu streicheln und dabei die Intensität der Handlungen im Verlauf stückweise erweitert habe. Herr Z. habe weiterhin angegeben, die Tathandlungen mit seiner Digitalkamera aufgenommen zu haben, um sie sich später jederzeit – z. B. auch wegen Stress oder nach beruflichen oder partnerschaftlichen Konfliktsituationen - wieder anschauen zu können.

Herr Z. habe sich das erste Mal in seiner Schulzeit (7. Klasse) in einen gleichaltrigen Mitschüler verliebt. In der Folgezeit habe er sowohl mit Frauen als auch mit Männern Affären und kürzere Beziehungen von bis zu 3 Jahren geführt, die beiderseitig als zufriedenstellend erlebt worden seien. Das Alter der männlichen Partner sei dabei im Verlauf rückläufig gewesen. Aktuell bevorzuge Herr Z. Jungen im Alter von 4-6 Jahren. Seit seiner Jugend habe er Pornografie homosexueller Natur genutzt, im zeitlichen Verlauf allerdings mehr und mehr Kinderpornografie.

Herr Z. stehe in regelmäßigen Kontakt mit seiner Primärfamilie, zudem habe er zwei beste Freunde, die er aus der gemeinsamen Schulzeit kenne. Einen quantitativen Anstieg der sexuellen Übergriffe zum Nachteil von Jonas habe es nach seiner Kündigung vor wenigen Wochen nicht gegeben. Er habe weiterhin von Kinderpornografie Gebrauch gemacht und dazu masturbiert (circa 5 Mal pro Woche). Die Kündigung habe zwar kurzzeitig am Selbstwert des Herrn Z. genagt, er sei sich aber sicher gewesen bald eine neue Anstellung zu finden.

Zur delinquenten Vorgeschichte: Herr Z. wurde im Alter von 27 Jahren beschuldigt, sich Jungen einer Schwimmmannschaft im Alter von 6-12 Jahren in sexueller Absicht genährt zu haben, das Verfahren wurde damals nach § 170 II StPO eingestellt.
