# Supplementary material for: Interrater reliability of the criteria-based assessment of criminal responsibility in paraphilic disorders
Source: Nervenarzt. 2020 May 11;92(1):1–8. [Article in German] doi: 10.1007/s00115-020-00920-1 (PMC7808990; doi:10.1007/s00115-020-00920-1)
Supplement: Supplementary file 2 [file 115_2020_920_MOESM2_ESM.docx]

Fallvignette 2:

Der Angeklagte Herr M. ist 24 Jahre alt (*05.02.1994). Ihm wird vorgeworfen, in den Abendstunden des 10.03.2018 gegen 20 Uhr die vorbeigehende 48-jährige Frau N. auf dem hell ausgeleuchteten Gehweg vor der Haustür seines Wohnhauses (ein Mehrfamilienhaus mit insgesamt 12 Mietparteien) von hinten gepackt, ihr mit der linken Hand den Mund zugehalten, mit dem rechten Arm die Oberarme fixiert und die sich wehrende Frau anschließend in seine, in der 1. Etage gelegene Mietwohnung verschleppt zu haben. In der Wohnung angekommen habe er der Frau, die fortlaufend um Hilfe geschrien habe, einen heftigen Schlag mit einem Hammer auf den Hinterkopf verpasst, so dass sie das Bewusstsein verloren habe. Dann habe er der Frau den Mund mit Paketklebeband zugeklebt und sie mit Kabelbindern an den Hand- und Fußgelenken gefesselt. Anschließend habe er sich vor der immer noch bewusstlosen Frau stehend 2-malig bis zum Samenerguss selbstbefriedigt. Als Frau N. nach ca. 3 Stunden wieder zu Bewusstsein gekommen sei, habe Herr M. den oralen Geschlechtsverkehr eingefordert, indem er sie mehrfach an den Haaren gepackt und ihr mehrere heftige Faustschläge gegen Hals und Ohren versetzt habe. Ein Nachbar, der nun auf das Schreien der Frau aufmerksam geworden sei, habe schließlich die Polizei alarmiert.

In der Beschuldigtenvernehmung der Kriminalpolizei gab Herr M. u. a. an, dass er bereits mehrere Stunden zuvor an seinem Fenster gestanden und Ausschau nach Frauen gehalten habe. Frau N. sei die erste Frau gewesen, die allein unterwegs gewesen sei. Dazu habe er an seinem Glied manipuliert und in Gedanken die gewalttätige Überwältigung einer ahnungslosen Frau vorfantasiert und im Internet nach sadistischen Pornos gesucht. Über Nachbarn, Passanten oder sonstige mögliche Störeinflüsse habe er sich keine Gedanken gemacht – „Ich hatte einen Tunnelblick“. Das Quälen und Erniedrigen der Frau in Form von Schlägen und an den Haaren reißen sei dabei mit einem besonderen sexuellen Lustgewinn verbunden gewesen.

Herr M. habe weiterhin angegeben, sich seit seinem 13. Lebensjahr regelmäßig Pornografie anzusehen. Es gebe Tage, an denen er im Internet Stunden lang (bis zu 5 Stunden) ihn stimulierende Inhalte suche – seine Vorliebe sei dabei Vergewaltigungssex mit Fesselungen im Verbund mit der Ausübung von körperlicher Gewalt und Demütigung. In Phasen von Stress und Zurückweisungen reagiere er sich verstärkt in Form von Selbstbefriedung ab, weil es ihm danach kurzfristig besser gehe. Ausgenommen einer 10 Monate andauernden Beziehung zu einer ehemaligen Schulkameradin, in der es wiederholt zu häuslicher Gewalt gekommen sei, habe er bislang keinerlei Partnerschaftserfahrungen. Herr M. besuche regelmäßig Prostituierte.

Bis auf seine ältere Schwester, die Herrn M. auch aktuell in der Untersuchungshaft regelmäßig besuche, habe er keine nennenswerten sozialen Kontakte. Im Vorfeld habe er sich lediglich mit einem älteren Herrn aus der gegenüberliegenden Wohnung zum Kartenspielen getroffen. Infolge eines Streits mit seinen Eltern, 14 Tage vor der mutmaßlichen Tat, hätten seine Eltern endgültig den Kontakt zu ihm abgebrochen. Grund hierfür sei gewesen, dass Herr M. das Vorhaben einer Berufsausbildung verworfen habe. Hier habe Herr M. ein weiteres Mal Ablehnung erfahren.

Zur delinquenten Vorgeschichte: Herr M. wurde vier Wochen vor der mutmaßlichen o. g. Tat aus einer Jugendhaftanstalt entlassen, wo er eine Jugendstrafe von 3 ½ Jahren wegen sexueller Nötigung in 2 Fällen und einem weiteren Fall der versuchten sexuellen Nötigung abgesessen hatte. Eine Therapie habe Herr M. abgelehnt, da er sich für psychisch gesund gehalten habe.
